# Ivonescimab combined with chemotherapy shows promising efficacy in an ALK fusion-positive lung adenocarcinoma patient with ALK-TKI resistance: a case report

**DOI:** 10.3389/fonc.2025.1723763

**Published:** 2025-12-18

**Authors:** Meiyuan Lin, Yuan Gao, Jun Deng

**Affiliations:** Department of Respiratory and Critical Care Medicine, The Affiliated Hospital of Southwest Medical University, Luzhou, Sichuan, China

**Keywords:** ivonescimab, non–small cell lung cancer, ALK fusion, drug resistance, BIM deletion polymorphism, case report

## Abstract

This report describes a case of lung adenocarcinoma harboring an echinoderm microtubule-associated protein-like 4–anaplastic lymphoma kinase (EML4–ALK) fusion, high programmed death-ligand 1 (PD-L1) expression, and a Bcl-2-like protein 11 (BIM) deletion polymorphism. The patient was initially treated with alectinib, but experienced rapid disease progression. After repeat genetic testing, therapy was switched to lorlatinib, which again resulted in early progression. Subsequently, treatment with ivonescimab combined with chemotherapy achieved a favorable clinical response. To our knowledge, this is the first reported case of ivonescimab use in such a patient. This case offers a potential reference for the management of ALK fusion–positive lung adenocarcinoma resistant to ALK tyrosine kinase inhibitors (ALK-TKIs).

## Introduction

Lung cancer poses a serious threat to human health and remains the leading cause of cancer-related deaths worldwide. Approximately 60% of patients with lung adenocarcinoma harbor driver gene alterations, and about 2%–7% of these cases exhibit anaplastic lymphoma kinase (ALK) fusions (1). For patients with advanced non–small cell lung cancer (NSCLC) harboring ALK fusions, targeted therapy has become the standard first-line treatment, including first-generation (e.g., crizotinib), second-generation (e.g., alectinib), and third-generation (e.g., lorlatinib) ALK tyrosine kinase inhibitors (ALK-TKIs) (2–5). Although the use of ALK-TKIs has significantly improved clinical outcomes in this population, the inevitable emergence of drug resistance remains a major clinical challenge (6). Therefore, identifying effective subsequent treatment strategies for ALK fusion–positive NSCLC patients who experience disease progression after ALK-TKI therapy has become a key focus of current research.

We report a rare case of ALK fusion–positive lung adenocarcinoma with concurrent high programmed death-ligand 1 (PD-L1) expression and a Bcl-2-like protein 11 (BIM) deletion polymorphism. The patient exhibited resistance to both second- and third-generation ALK-TKIs but achieved a sustained clinical response to ivonescimab combined with chemotherapy. This case provides new insights into personalized treatment strategies for ALK-TKI–resistant NSCLC.

## Case presentation

**Figure 1:**
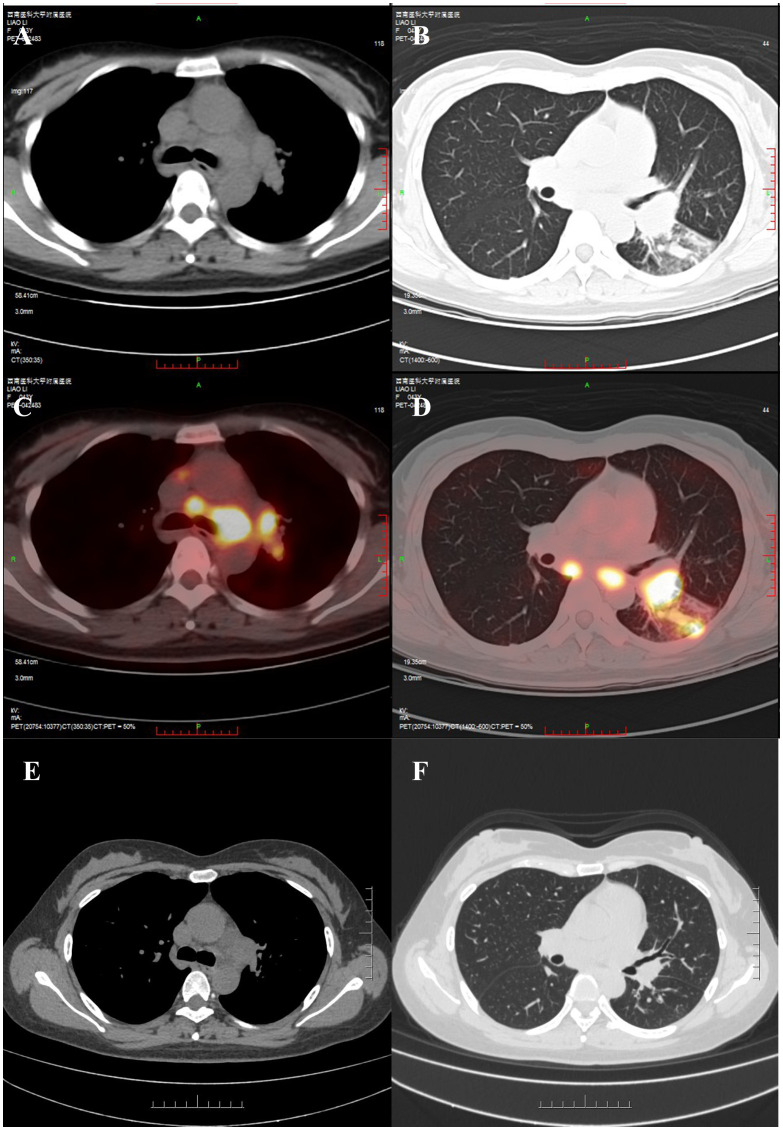
Initial imaging revealed a left lower hilar mass with increased glucose metabolism (measuring approximately 3.5 cm × 2.5 cm in maximum cross-section), along with enlarged and hypermetabolic mediastinal and hilar lymph nodes. A solid nodule in the dorsal segment of the left lower lobe also exhibited hypermetabolism. No distant metastases were identified. **(A, B)** Chest CT; **(C, D)** PET-CT. **(E, F)** Follow-up imaging on January 4, 2024, showed significant regression of the lesions in the left hilum and the dorsal segment of the left lower lobe, as well as a decrease in size of some mediastinal lymph nodes.

On June 5, 2023, a 43-year-old woman presented to the Pulmonology Department with cough and sputum production. She had no history of smoking or other comorbidities. Her vital signs were stable. Bilateral supraclavicular lymphadenopathy was noted. Chest CT and FDG PET-CT suggested lung cancer with possible lymph node and intrapulmonary metastases (**Figures 1A–D**). Biopsies of bilateral supraclavicular lymph nodes and lung tissue confirmed lung adenocarcinoma with lymph node involvement, leading to a diagnosis of stage IIIC (T3N3M0) lung adenocarcinoma. On June 27, 2023, genetic analysis of the tissue specimen by next-generation sequencing revealed an EML4 exon 10–ALK intron 19 fusion (allele frequency: 6.43%) and a BIM deletion polymorphism in intron 2. High PD-L1 expression was also observed, with a tumor proportion score (TPS) of 75%. Because the patient declined chemoradiation, she was started on alectinib (600 mg, twice daily) as first-line therapy, achieving a partial response (**Figures 1E,F**) without significant adverse events.

**Figure 2:**
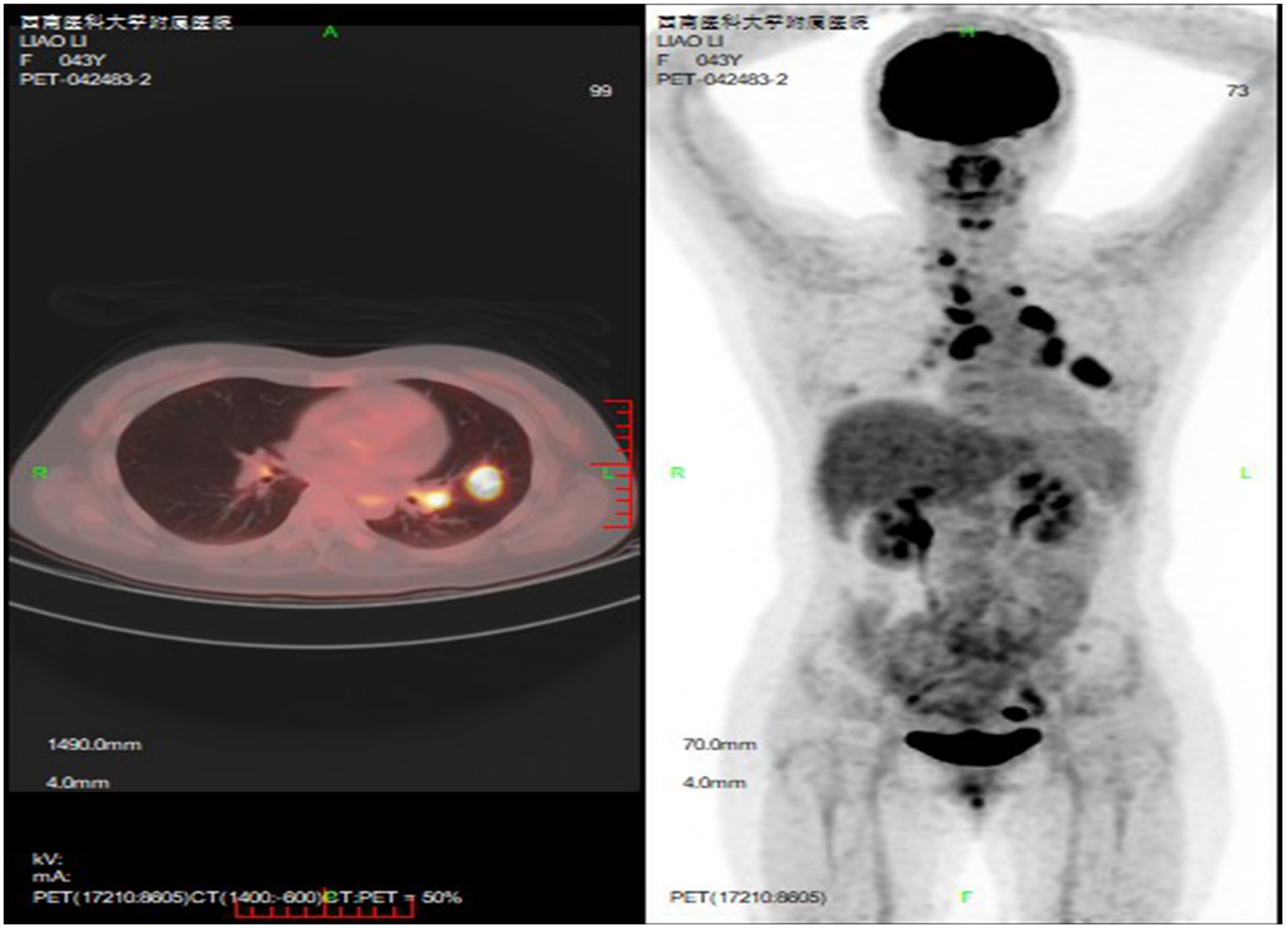
A newly developed mass was identified in the lingular segment of the left upper lobe, measuring approximately 3.6 cm × 2.8 cm in maximum cross-section, with associated increased glucose metabolism (SUVmax ≈ 8.2). Multiple enlarged hypermetabolic lymph nodes (SUVmax ≈ 12.0) were present in the right supraclavicular fossa, mediastinum, and bilateral hilar regions. There was no evidence of distant metastasis.

**Figure 3:**
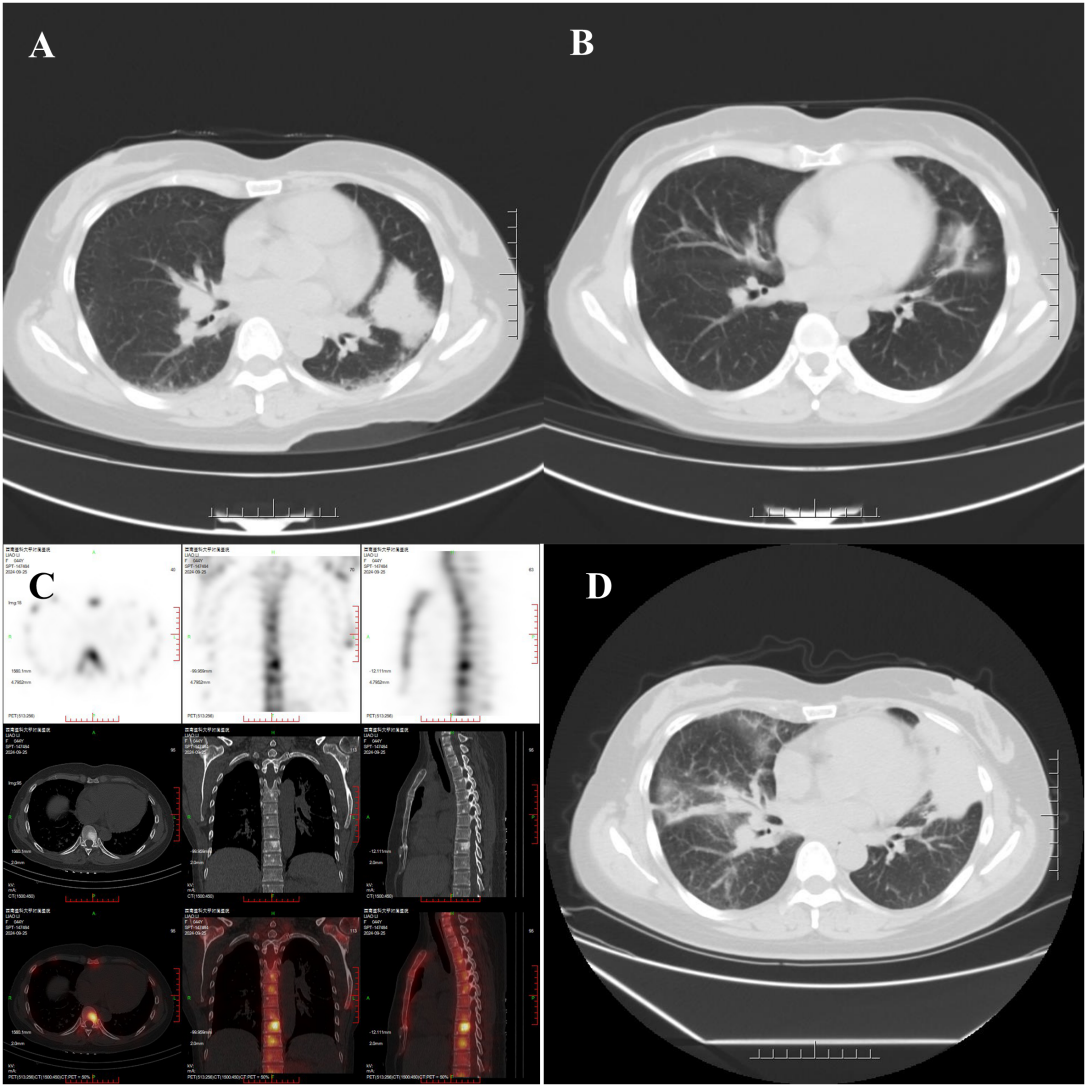
**(A)** CT on May 7, 2024, demonstrated enlargement of the lingular mass to 4.3 × 3.2 cm, along with an increase in size of multiple scattered solid nodules in both lungs. **(B)** CT on September 23, 2024, showed a reduction in the size of the lingular mass to 2.9 × 1.2 cm. **(C)** Whole-body bone scintigraphy revealed increased metabolic activity in parts of the vertebrae and bilateral ribs. **(D)** CT on December 28, 2024, indicated re-enlargement of the lingular mass to 6.7 × 3.5 cm, occlusion of the bronchus in the lower lingular segment of the left upper lobe, and multiple scattered nodular, patchy, and cord-like high-density opacities in both lungs.

On February 21, 2024, FDG PET/CT revealed a new lesion in the lingular segment of the left upper lobe (**Figure 2**), along with enlarged lymph nodes in the right supraclavicular fossa, mediastinum, and bilateral hilar regions, with no evidence of distant metastasis. Due to extensive lymph node involvement, radiotherapy was not performed. Bevacizumab (15 mg/kg, every 3 weeks) was added to her regimen for three cycles. On May 7, 2024, CT re-evaluation demonstrated disease progression (**Figure 3A**). The needle biopsy of the left upper lung lesion revealed lung adenocarcinoma. Subsequent genetic analysis by next-generation sequencing identified an EML4 exon 6–ALK exon 20 fusion (allele frequency: 11.2%) and an ALK G1202R mutation (allele frequency: 25.0%). Meanwhile, immunohistochemistry showed that PD-L1 expression remained high (TPS 98%). Therapy was switched to lorlatinib (100 mg, once daily), resulting in tumor shrinkage (**Figure 3B**), though bone metastases were detected (**Figure 3C**). After 7 months of lorlatinib, bone pain developed. On December 28, 2024, CT showed disease progression (**Figure 3D**). Lorlatinib was discontinued, and considering her high PD-L1 expression, she was treated with pembrolizumab (200 mg) along with denosumab for bone protection. She subsequently underwent palliative radiotherapy for bone pain at an external hospital.

**Figure 4:**
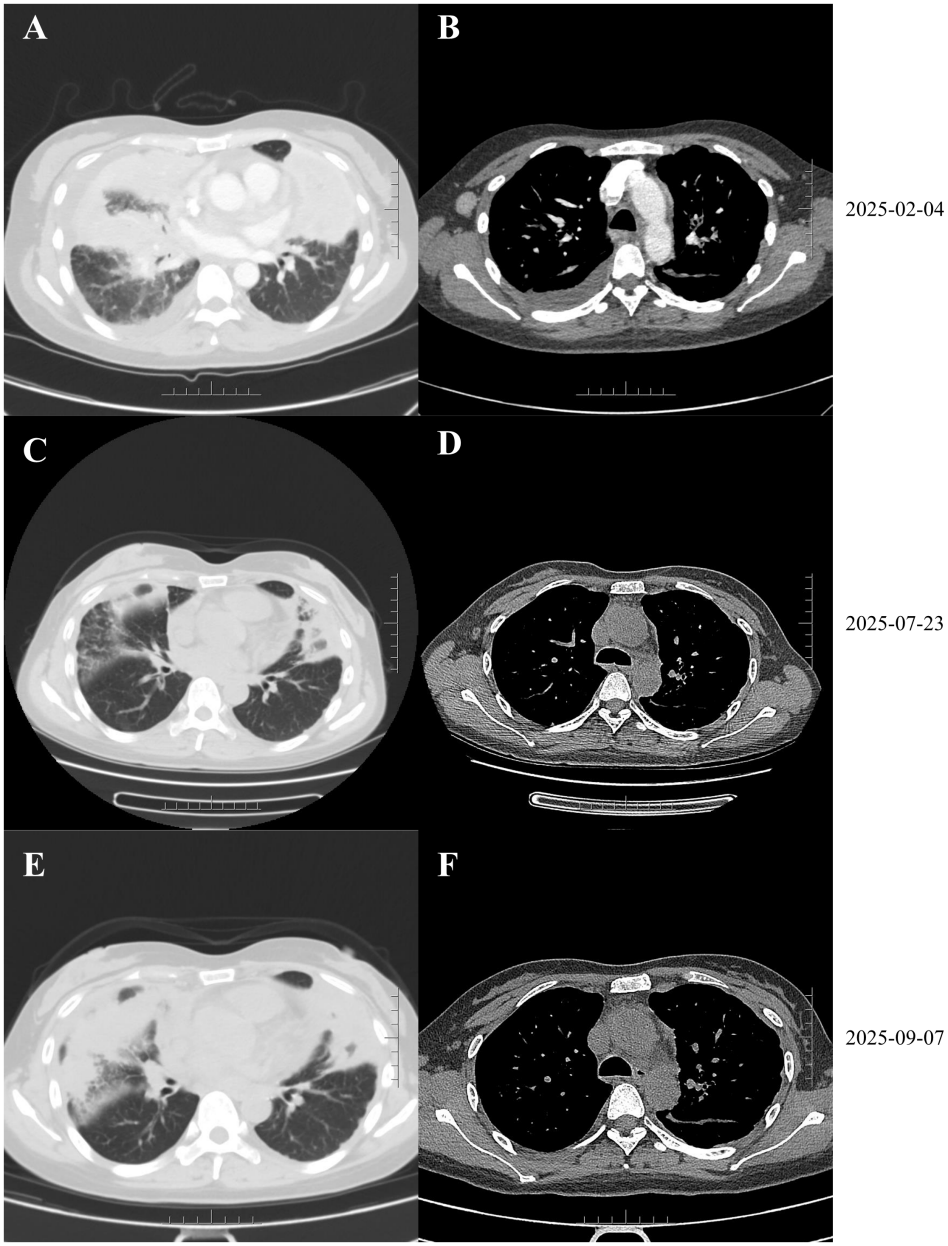
**(A, B)** The mass in the lingular segment of the left upper lobe further enlarged to 8.4 × 4.4 cm. This was accompanied by multiple nodular, patchy, and cord-like high-density opacities in both lungs and enlarged right axillary lymph nodes. **(C–F)** Follow-up CT scans obtained during treatment with ivonescimab demonstrated a reduction in the size of both the pulmonary lesions and lymph nodes compared to pre-treatment imaging.

On February 4, 2025, CT revealed tumor enlargement to 8.4 × 4.4 cm, increased pulmonary nodules, and enlarged right axillary lymph nodes (**Figures 4A, B**). A new right breast mass was detected, and fine-needle aspiration cytology confirmed metastatic adenocarcinoma. After discussion with the patient and her family, treatment with ivonescimab (20 mg/kg, every 3 weeks) combined with pemetrexed (500 mg/m²) and carboplatin (AUC = 5) was initiated on February 8, 2025. She received 6 cycles of ivonescimab, pemetrexed, and carboplatin, followed by maintenance ivonescimab and pemetrexed. As of September 23, 2025, she had completed 10 cycles of ivonescimab plus chemotherapy, spanning over 7 months, resulting in tumor shrinkage and reduction of right axillary lymph nodes (**Figures 4C–F**). No severe adverse events were observed during treatment.

## Discussion

This report presents a complex case of advanced lung adenocarcinoma. The patient harbored an EML4–ALK gene fusion, high PD-L1 expression, and a BIM deletion polymorphism, and later developed resistance to ALK-TKI therapy. The clinical course of this case highlights the challenges associated with precision therapy in advanced NSCLC.

The ALK gene is a common oncogenic driver in NSCLC, with EML4-ALK being the most prevalent fusion form (7). With advances in targeted therapy, multiple generations of ALK-TKIs have been approved for ALK fusion–positive NSCLC. First-generation TKIs significantly improved survival compared with chemotherapy but were limited by poor central nervous system (CNS) penetration and the early emergence of resistance (8). Second-generation TKIs exhibit enhanced CNS penetration and significantly prolong progression-free survival, establishing them as the current standard first-line treatment (3). Third-generation TKIs retain activity against several known resistance mutations and represent an important option after failure of second-generation TKIs (9). Nevertheless, ALK-TKI resistance remains a persistent challenge. Resistance mechanisms fall into two primary categories: on-target and off-target. On-target resistance primarily involves secondary mutations in the kinase domain, such as G1202R, which reduces drug-binding affinity and consequently decreases TKI efficacy. Off-target resistance includes bypass pathway activation; for example, EGFR-mediated bypass signaling can reactivate downstream pathways and ultimately promote treatment resistance (10).

ALK fusion–positive is predominantly observed in young, female, non-smoking patients with adenocarcinoma (2), consistent with the characteristics of this patient. Clinically, EML4–ALK is the most frequent ALK rearrangement. This patient received the second-generation ALK-TKI alectinib as first-line therapy; however, the clinical benefit lasted only 8 months, markedly shorter than the median progression-free survival (PFS) reported in clinical trials (11). This suggests the presence of early acquired resistance. In epidermal growth factor receptor (EGFR)-mutant NSCLC, combining bevacizumab with erlotinib significantly improves PFS (12, 13). Similarly, small-sample studies have evaluated the combination of ALK-TKIs with bevacizumab in ALK fusion–positive NSCLC. Case reports have suggested that bevacizumab may overcome early alectinib resistance through enhanced tumor drug delivery (14). Clinical studies further confirm that adding bevacizumab to alectinib is safe, improves PFS, and may delay the emergence of resistance in ALK-rearranged NSCLC (15, 16). Unfortunately, in this patient, the addition of bevacizumab after resistance had developed did not result in significant improvement. This indicates that in the context of a strong driver oncogene, angiogenesis inhibition alone may be insufficient to overcome ALK pathway–mediated resistance. The detection of the ALK G1202R mutation on the second round of genetic testing marked a key turning point in the patient’s treatment course. G1202R is the most common secondary resistance mutation following treatment with second-generation ALK-TKIs. Among patients who develop resistance to brigatinib, alectinib, and ceritinib, the incidence of G1202R has been reported as 43%, 29%, and 21%, respectively (9). This mutation is insensitive to first- or second-generation ALK-TKIs but remains responsive to the third-generation inhibitor lorlatinib. The findings in this case are fully consistent with this understanding, providing a solid rationale for switching to lorlatinib. EML4–ALK variants influence secondary resistance, as different breakpoints generate distinct fusion protein forms. The most common variants are variant 1 (E13;A20) and variant 3 (E6;A20) (17), with variant 3 being associated with early disease progression during alectinib treatment (18). Notably, the G1202R mutation occurs more frequently in variant 3 than in variant 1 (19, 20). These findings suggest that determining ALK variants should be part of the initial diagnostic workup for NSCLC, as it may guide the selection of the appropriate ALK-TKI. In this patient, genetic testing revealed variant 3 carrying the G1202R mutation, indicating that third-generation ALK-TKI therapy may be more suitable as first-line therapy.

In addition, this patient harbored a BIM deletion polymorphism in intron 2, which may have contributed to the suboptimal efficacy of ALK-TKIs. BIM, a member of the BCL2 family of proteins, plays a crucial role in regulating apoptosis in lung cancer cells (21). The BIM deletion polymorphism involves a 2903-bp deletion that results in the loss of the pro-apoptotic BCL2 homology domain 3 (BH3) in BIM isoforms, thereby inhibiting tumor cell apoptosis (22). Numerous studies have shown that in NSCLC patients with EGFR mutations, BIM deletion polymorphism is associated with poor response to EGFR-TKI therapy (22–24). A similar effect appears to occur in ALK fusion–positive patients. Zhang et al. reported that among ALK fusion–positive NSCLC patients treated with crizotinib, those with BIM deletion polymorphism had significantly shorter PFS and lower objective response rates (25). Hou et al. found that downregulation of BIM increased resistance to alectinib in NSCLC (26). In this case, even after switching to lorlatinib, a third-generation ALK-TKI capable of overcoming the G1202R mutation, the patient’s PFS was only 4 months. Unfortunately, a third genetic test was not performed. This phenomenon suggests that BIM deletion polymorphism may act as a persistent background factor influencing TKI efficacy, independent of secondary ALK resistance mutations. For NSCLC patients with driver gene positivity and BIM deletion polymorphism, BH3 mimetics may potentially prolong PFS with ALK-TKI therapy.

Research indicates that EML4-ALK fusion proteins and EGFR activation can upregulate PD-L1 expression, promoting tumor immune evasion (27–29). Although preclinical studies suggest that PD-1 blockade may inhibit TKI-resistant tumor cells (27, 29), clinical evidence remains limited. Case reports by Yamasaki et al., Shimada et al., Baldacci et al., and Matsumura et al. have documented responses to PD-1 inhibitors in ALK-positive patients following TKI failure, particularly those with high PD-L1 expression and TKI resistance (30–33). A retrospective study further indicated that some patients with PD-L1–positive expression may benefit from immunotherapy-based treatment after the development of resistance to ALK-TKIs (34). In our case, pembrolizumab showed no therapeutic response, despite a PD-L1 expression of 98% after ALK-TKI resistance. This aligns with previous reports indicating the limited efficacy of PD-1/PD-L1 inhibitors in oncogene-driven NSCLC (35). Notably, elevated PD-L1 expression in ALK fusion–positive NSCLC patients has been associated with poorer outcomes, suggesting that it may function more as a prognostic biomarker (36). Additionally, combining TKIs with immune checkpoint inhibitors (ICI) has been associated with a significantly increased incidence of treatment-related adverse events (1). Given the currently limited evidence, the therapeutic value of ICI in ALK fusion–positive NSCLC patients with different clinical and molecular characteristics warrants further investigation.

Ivonescimab, a first-in-class humanized bispecific antibody targeting both programmed cell death protein 1(PD-1) and vascular endothelial growth factor A (VEGF-A), enhances therapeutic efficacy in NSCLC by simultaneously inhibiting PD-1/PD-L1–mediated immune escape and VEGF-driven angiogenesis. In the presence of VEGF, ivonescimab’s binding affinity to PD-1 is increased, strengthening PD-1/PD-L1 signal blockade. Conversely, PD-1 enhances ivonescimab’s binding to VEGF, further augmenting VEGF signal inhibition (37). Compared with combinations of antiangiogenic agents and PD-1/PD-L1 inhibitors, ivonescimab simultaneously binds to PD-1 and VEGF, with mutual interactions enhancing the biological effect and achieving a “1 + 1 > 2” outcome (38). Additionally, ivonescimab eliminates Fc effector functions, abolishing antibody- and complement-dependent cytotoxicity and improving clinical safety (39). Ivonescimab combined with pemetrexed and carboplatin has been approved for locally advanced or metastatic EGFR mutation–positive NSCLC patients with disease progression after EGFR-TKI therapy (40); however, its use in ALK-TKI–resistant, ALK fusion–positive NSCLC has not been reported. One study enrolling 322 patients with locally advanced or advanced NSCLC who progressed during EGFR-TKI therapy reported a median PFS of 7.06 months in the ivonescimab plus chemotherapy group versus 4.08 months in the chemotherapy-only group (41). Another study in stage IIIB/IV NSCLC patients with EGFR mutations or ALK/ROS1 fusions who progressed after TKI therapy compared two regimens: one adding bevacizumab to platinum, pemetrexed, and atezolizumab, and the other using platinum, pemetrexed, and atezolizumab alone. The study reported median PFS of 7.3 and 7.2 months, respectively (42). To date, in this case, following ALK-TKI resistance, the patient has received ivonescimab combined with chemotherapy for 7 months, with disease remaining stable. This suggests that, after developing resistance to ALK-TKIs, ivonescimab may serve as a potential therapeutic option for NSCLC patients who are ALK fusion–positive, have high PD-L1 expression, and carry a BIM deletion polymorphism.

The diagnostic and therapeutic course of this case provides several important insights. First, it underscores the central role of dynamic and comprehensive biomarker assessment in guiding treatment throughout the disease course, encompassing not only driver genes and resistance mutations but also key molecules such as BIM that influence therapeutic response. Second, it demonstrates that high PD-L1 expression has limited predictive value for ICI monotherapy in ALK-positive lung cancer. Finally, this is the first report demonstrating the significant efficacy of ivonescimab combined with chemotherapy in ALK-TKI–resistant patients, offering an individualized framework for clinical management of such complex cases. Future prospective studies are warranted to validate the role of bispecific antibodies in later-line therapy for ALK fusion–positive NSCLC and to explore optimal sequential or combination strategies with targeted agents.

## Data Availability

The original contributions presented in the study are included in the article/Supplementary Material. Further inquiries can be directed to the corresponding author.
